# Development of Synthetic and Natural Materials for Tissue Engineering Applications Using Adipose Stem Cells

**DOI:** 10.1155/2016/5786257

**Published:** 2016-02-10

**Authors:** Yunfan He, Feng Lu

**Affiliations:** Department of Plastic and Cosmetic Surgery, Nanfang Hospital, Southern Medical University, Guangzhou, Guangdong 510515, China

## Abstract

Adipose stem cells have prominent implications in tissue regeneration due to their abundance and relative ease of harvest from adipose tissue and their abilities to differentiate into mature cells of various tissue lineages and secrete various growth cytokines. Development of tissue engineering techniques in combination with various carrier scaffolds and adipose stem cells offers great potential in overcoming the existing limitations constraining classical approaches used in plastic and reconstructive surgery. However, as most tissue engineering techniques are new and highly experimental, there are still many practical challenges that must be overcome before laboratory research can lead to large-scale clinical applications. Tissue engineering is currently a growing field of medical research; in this review, we will discuss the progress in research on biomaterials and scaffolds for tissue engineering applications using adipose stem cells.

## 1. Introduction

Adipose stem cells (ASCs) have the potential to differentiate into various cell phenotypes if there is a specific inducing microenvironment using suitable inductive substances [1–3]. Meanwhile, their abundance and relative ease of harvest, along with their autogenous immune-privileged status, have also made them an attractive candidate for tissue engineering and regenerative therapies [4, 5].

Tissue engineering enables the regeneration or repair of tissues and organs through combinations of stem cells, biomaterial scaffolds, and regulatory growth factors [6, 7]. ASC-based tissue engineering strategies depend primarily on the quality of the ASC fraction used. Although a large number of studies have been conducted to assess the differentiation potential of ASCs in different carriers, there are still some unclear aspects regarding the basic knowledge of ASC biology and the clinical applications. These include the following: (i) What is the best definition for ASCs, and what is the true nature of the fractions of ASCs used by the various investigators? (ii) Does heterogeneity exist between freshly isolated ASCs and ASCs expanded in several culture passages? (iii) What are the best procedures for harvesting adipose tissue, preparing the stromal vascular fraction (SVF), and isolating ASCs? (iv) What are the best procedures for cell banking and cellular cryopreservation? The past few years have seen exciting progress in tissue engineering and regenerative medicine using various biomaterials and scaffold [8–11]. Natural and synthetic materials have been developed to provide a carrier scaffold that is ideally supposed to mimic the extracellular matrix (ECM) properties of an* in vivo* microenvironment to induce tissue formation [12, 13]. Nowadays, the development of efficient biomaterials and scaffolds is still in high demand for the production of clinically useable volumes of new tissues to replace lost or malfunctioning body parts and to achieve uncomplicated wound healing.

It is crucial that scaffolding materials can positively interact with surrounding tissue to not only fill the defect, but also facilitate the natural regeneration of stem cells. Significant efforts have been made to develop such scaffolds for tissue engineering applications [14–16]. For example, electrospinning, lithography, microfabrication, and self-assembly techniques have been widely explored for the fabrication of engineered scaffolds appropriate for specific tissue applications. Considering the usage of these engineered scaffolds in the body, they should have the following characteristics: (i) possession of appropriate surface properties to promote the adhesion, proliferation, and differentiation of stem cells; (ii) low toxicity and immunogenicity; (iii) high porosity; and (iv) degradability that is adequate for specific tissues, with an interconnected pore network for cell growth and flow transport of nutrients and metabolic waste. The objective of this review is to recapitulate the progress in the fields of biomaterial and scaffold development and various procedures for ASC selection for tissue engineering applications and to review several clinical cases for the advancement of ASCs-based tissue engineering strategies.

## 2. Basic Knowledge on the Biology of ASCs in Regenerative Medicine

In the past decade, a number of cell characterization studies have described the underlying biology of ASCs [17–33]. Preclinical studies on the use of ASCs both* in vitro* and* in vivo* have been performed, and the efficacy of ASCs has been determined in several clinical trials [34–40]. Compared with bone marrow or umbilical cord stem cells, ASCs have a similar self-renewal ability* in vitro*, and the ability of ASCs to differentiate in other mesodermal and ectodermal lineages has been demonstrated on several occasions [1, 41–45]. Moreover, ASCs release multiple growth factors, such as the two key factors vascular endothelial growth factor (VEGF) and hepatocyte growth factor (HGF). Other factors include VEGF-B, VEGF-C, fibroblast growth factor- (FGF-) 2, angiopoietin- (Ang-) 1, Ang-2, SPARC/osteonectin, platelet-derived growth factor- (PDGF-) b, transforming growth factor (TGF), and stromal cell-derived factor-1 (SDF-1) [46]. However, the specific population of ASCs with the greatest therapeutic potential remains unclear. Since the initial reports in the late 1960s [47], many researchers have established that stromal stem cells similar to those identified in bone marrow can be isolated from adipose tissue that is either resected as intact tissue or aspirated using tumescent liposuction [17, 48, 49]. In general, the obtained adipose tissue is digested with one of the following: collagenase, dispase, trypsin, or related enzymes. A consensus exists regarding temperature (37°C), digestion duration times (range, 30 min to >1 h), and ratios of tissue weight to volume; however, protease concentrations are far more variable. Following the neutralization of the enzymes and differential centrifugation, the released elements, which are separated from the mature adipocytes, are defined as the SVF. The SVF consists of a heterogeneous mesenchymal population of cells that includes not only adipose stromal and hematopoietic stem and progenitor cells but also endothelial cells, erythrocytes, fibroblasts, lymphocytes, monocyte/macrophages, and pericytes [17, 50, 51]. For the phenotypic characterization of the SVF, the International Federation for Adipose Therapeutics and Science (IFATS) and the International Society for Cellular Therapy (ISCT) proposed a stromal cell population, excluding hematopoietic and endothelial cells, based on the following combination: CD45−CD235a−CD31−CD34+ and additional markers used to identify SVF are CD13 (APN), CD73 (L-VAP-2), CD90 (Thy-1), and CD105 (Endoglin). Based on the existing literature, this population combination represents at least 20% of the cells in the SVF [52–56], and the percentage of CD34+ cells mainly depends on the method used to harvest the adipose tissue, the degree of vascular hemorrhage, and the subsequent digestion and isolation techniques. In addition to the enzyme digestion, methods for isolating SVF cells using mechanical, nonenzymatic techniques have been developed recently, and some have been applied in clinical practice [57–59].

When SVF pellets are seeded into culture, a subset of elongated cells begins to adhere to the bottom of the plastic tissue culture plate. After a combination of washing steps and culture expansion with media to remove most of the hematopoietic cell population from the SVF cells, these cells are purified as an adherent cell population termed ASCs. ASCs are less heterogeneous than SVF cells and have the ability to undergo self-renewal and the capacity to undergo multilineage differentiation and generate multiple terminally differentiated cells when cultured in specific lineage-inducing culture media. One main difference between SVF cells and ASC suspensions is the high percentage of CD45+ cells in the SVF cell population (30–70%) and the low or undetectable percentage in ASC population (2–30%). ASCs generally express CD34+ during the early phase of culture (within 8–12 population doublings after culture of the SVF), but then its expression decreases with continued cell division [51, 55]. A joint statement by the IFATS and ISCT recommended that the surface antigens used to characterize ASCs should include CD73, CD90, and CD34 without CD45 and CD31. In addition, CD13 has also been proposed as an alternative or supplement to CD105 [17]. To date, most experimental research groups have isolated ASCs by tissue digestion, centrifugation, and the capacity of ASCs to adhere to cell culture plastic surfaces [9, 43, 60]. However, the adherent cell population also contains other cell types that are not multipotential [61–63]. In order to overcome the problem of “contamination,” a number of alternative methods have been proposed, including magnetic-activated cell sorting (MACS) and fluorescence-activated cell sorting (FACS). FACS is a typical cell enrichment method that utilizes complementary fluorochrome conjugated antibodies to label cells of interest. However, the sorted cells obtained from FACS can be utilized for diagnostic and experimental purposes but not for therapeutics due to problems with safety and efficacy [64, 65]. MACS is an antibody-aided technique based on immunomagnetic beads coated with specific antibodies against stem cell surface molecules, and it is technically accessible and affordable. From the view of clinical application, MACS with biodegradable magnetic beads wins over FACS on the grounds of safety, and it is the only method approved for use in clinical settings [61, 66–68].

Clinical research on adult stromal cell populations has accelerated, and multiple clinical investigations are underway to examine the use of ASCs and SVF cells for tissue engineering and regenerative medical applications [22, 69, 70]. To achieve the large numbers of ASCs required for clinical applications, either the cells need to be expanded in culture or ASCs must be pooled from multiple donors. Therefore, the development of stem cell banks is necessary. These banks must assure the quality and safety of these cell products, especially when the stored ASCs are intended for clinical use in cell therapy and regenerative medicine. Cryopreservation may be an ideal option for this and is currently the only method to preserve ASCs while maintaining their functional properties and genetic characteristics in the long term [71–73]. Slow freezing and vitrification are the currently available methods for the cryopreservation of stem cells in laboratories and clinics [74–78]. Vitrification only works well with the cryopreservation of human cells in small volumes, such as oocytes, but it is ill-suited for large volumes of ASCs [79, 80]. Moreover, vitrification techniques require higher cryoprotectant agent concentrations, which induces toxicity and osmotic stress in cells and tissues. Slow freezing is an established technique pioneered in the early 1970s and involves cryopreserving biological samples at controlled freezing rates to avoid intracellular ice formation and minimize structural damage to the cell membrane and cytoskeleton [81, 82]. Cryoprotectant agents are used at relatively low concentrations [21] in slow freezing, which has become the standard method for cell and tissue cryopreservation. However, formation of ice crystals, extreme hyperosmolarity, and dehydration are still reported when cells undergo the slow freezing process. Dimethyl sulfoxide (DMSO) is the most widely used cryopreservant for cells, but it is known to be toxic at room temperature. Trehalose is a nontoxic disaccharide of glucose that may stabilize and preserve cells and cellular structures during the freezing procedure. A cryopreservation method using trehalose as a cryoprotective agent is recommended for the long-term preservation of ASCs compared to simple cryopreservation or to cryopreservation using DMSO alone. Other cryoprotective agents such as polyvinylpyrrolidone and methylcellulose have been developed to replace DMSO; however, they are less efficient than DMSO in terms of maintaining ASC viability [75].

Adipose-derived stem cells are a promising cell source for regenerative medicine. It is important to understand the basic knowledge and biology behind stem cells, and further research is needed to guarantee the safety of ASCs and the effectiveness of tissue engineering using ASCs.

## 3. Advancement in Synthetic Materials for Tissue Engineering Applications Using ASCs

In the provision of an appropriate microenvironment for cellular components to interact with, the extracellular matrix (ECM) is an important component of normal tissue that must be considered. Various synthetic materials have been developed to provide carrier scaffolds that mimic ECM properties for tissue regeneration and reconstruction in combination with ASCs (Table 1). The advantages of synthetic materials and scaffolds rely on the technical possibility that chemical and physical properties (e.g., porosity, surface characteristics, and degradation products nature) can be specifically optimized for a particular application [83, 84]. Ideally, a polymeric material used for tissue engineering should be able to regulate cell proliferation without the loss of pluripotency and to direct differentiation into a specific cell lineage when desired. Tan et al. described the influence of TiO_2_ nanofibrous surface structures, which were produced in situ onto Ti-6Al-4V substrate via a thermal oxidation process, on the regulation of proliferation and preservation of stemness of ASCs. The results show that ASCs exhibit better adhesion and significantly enhanced proliferation on TiO_2_ nanofibrous surfaces than on flat control surfaces, thus presenting a promising potential for the application of TiO_2_ nanofibrous surfaces in the field of bone tissue engineering and regenerative therapies [85]. Although much has been done to develop tissue-engineered skin substitutes in the past decade, poor visualization, hypertrophic scarring, and keloid formation are still possible negative outcomes for current skin graft strategies [86–88]. In an effort to overcome these limitations, Zonari et al. proposed the combination of poly(3-hydroxybutyrate-co-hydroxyvalerate) (PHBV) structures with ASCs to induce skin regeneration in a full-thickness model. In this work, PHBV scaffolds demonstrated good integration with the surrounding tissue, allowing exudation and infiltration by inflammatory cells, which may contribute to rapid degradation over time. Furthermore, PHBV scaffolds offered a moist environment combined with a stiff character that withstands contraction and simultaneously stimulates the secretion of various growth factors by seeded ASCs; these factors enhance vascularization and ECM deposition with reduced scarring. Ultimately, this study revealed the great advantages of PHBV loaded with ASCs to improve wound healing and skin regeneration with reduced scarring in skin tissue engineering [10]. The advancement of tissue engineering as a regenerative therapy relies on rapid vascularization of tissue constructs, and engineered three-dimensional biomaterials are known to affect the angiogenic capacity of seeded stem cells [89–91]. Copolymer PEGylated fibrin (P-fibrin) gels were introduced by Chung et al. as an ASC-carrying scaffold for encouraging local angiogenesis in an* in vitro* culture model without added soluble factors. In P-fibrin gels, ASCs elicited higher von Willebrand factor expression than the two commonly used hydrogels (i.e., collagen and fibrin). After seven days of cultivation, vascular endothelial growth factor (VEGF) was secreted more in fibrin and P-fibrin gels than in collagen; several other angiogenic and immunomodulatory cytokines were similarly enhanced. Moreover, P-fibrin matrices were uniquely able to drive a vessel-like phenotype in ASCs and induce formation of well-organized vascular networks relative to other gels. Thus, it can be speculated that the research on ASCs' regenerative potential in a carrier scaffold can be expanded to include cardiovascular and skin tissue engineering applications based on the observed angiogenic properties of ASCs in P-fibrin [91]. Seeding cells on mechanically appropriate scaffolds and applying specific mechanical stimulation to these cells have been found to be beneficial in terms of proliferation and differentiation [92, 93]. Frydrych et al. reported a large and flexible 3D porous poly(glycerol sebacate) (PGS)/poly(L-lactic acid) (PLLA) blend scaffold with mechanical properties comparable to adipose tissue that was fabricated via a freeze-drying and a subsequent curing process.* In vitro* cell test results provided clear evidence that PGS/PLLA scaffolds are suitable for the culture of ASCs, as they are characterized by deep cell penetration and ECM growth. This work demonstrates that the PGS/PLLA scaffolds provided favorable porous microstructures, good hydrophilic characteristics, and appropriate mechanical properties for soft tissue applications [93]. Neural tissue possesses a very limited capacity to regenerate new functional neurons after nerve injuries, and tissue-engineered neural tissues using stem cells may serve as a promising alternative for neural regeneration. However, such stem cells would need to proliferate and differentiate into the desired phenotype with the aid of adequate chemical, mechanical, or biological stimuli regeneration [94–96]. Catalpol is a natural active ingredient extracted from a traditional Chinese medicine. Guo et al. evaluated the effects of a catalpol-loaded scaffold on guiding the neuronal differentiation of hASCs. In their study, the process for catalpol loading into the electrospun poly(lactic-coglycolic acid)/multiwalled carbon nanotubes/silk fibroin (PLGA/MWCNTs/SF) nanofibrous scaffolds was successfully established. As a result of adding catalpol, the diameters of the nanofibers decreased and the porosity increased. Moreover, the mechanical properties of the composite scaffolds were improved, and more neuronal-like cells were found on scaffolds with catalpol [95]. The poor self-healing ability of cartilage necessitates the development of methods for cartilage regeneration. Fabrication of scaffolds with live stem cell incorporation and subsequent differentiation presents a promising route [97, 98]. Sun et al. [99] reported the use of a visible-light-based PSL (VL-PSL) system to encapsulate hASCs into a biodegradable polymer [poly-D,L-lactic acid/polyethylene glycol/poly-D,L-lactic acid (PDLLA-PEG)]/hyaluronic acid (HA) matrix to produce live cell constructs with customized architectures. In the chondrogenic medium-treated group (TGF-*β*3 group), hASCs showed high viability (84%) and expressed the chondrogenic genes Sox9, collagen type II, and aggrecan at 11, 232, and 2.29 × 10^5^ fold increases, respectively, compared to levels at day 0 in nonchondrogenic medium. After 28 days, the mechanical strength of the TGF-*β*3 group remained high at 240 kPa. Thus, PSL and PDLLA-PEG/HA-based fabrication method using ASCs is a promising approach for producing mechanically competent engineered cartilage.

Thus, synthetic materials provide greater control over the mechanical and biochemical properties of the carrier scaffolds and represent a promising tool in tissue engineering and regeneration medicine [100].

## 4. Development of Natural Materials for Tissue Engineering Applications Using ASCs

In accordance with the plastic surgery rule of “replace with alike,” natural materials have recently been recognized as an attractive choice for tissue engineering applications. Natural materials chosen for tissue engineering scaffolds are either compounds of the native ECM or polymers extracted from other biological systems [12, 101]. Evidence indicates that natural materials can behave similar to the ECM and possess biocompatibility, biodegradability, and inherent biological functions that could make them suitable for a range of tissue engineering applications [102–105]. Over the past several years, a wide range of natural materials has become available for tissue engineering strategies (Table 2). Among them, decellularized extracellular matrix has received increasing attention [106–110]. During tissue decellularization, cells are discharged from tissues, but the native ultrastructure and composition of the ECM is highly preserved, which is expected to be able to direct the differentiation fate of the seeded stem cells [111]. The combined use of decellularized human adipose tissue extracellular matrix (hDAM) and human adipose-derived stem cells (hASCs) as an adipose tissue engineering strategy was first introduced by Wang et al. [12]. In this study, engineered fat grafts (hDAM combined with hASCs) were implanted subcutaneously in nude rats. The results showed that hASCs seeded in hDAM contributed to adipose tissue formation; the implanted engineered fat grafts maintained their volume for eight weeks. Hence, this study provides a platform and novel scaffold design for adipose tissue engineering of hDAM-hASC constructs. Current cartilage tissue engineering technology has developed quickly and efforts have focused on the creation of a suitable chondrocyte scaffold. Acellular cartilaginous matrix (ACM), which is obtained from fresh cartilage using a series of acellular manipulations, is a recently developed natural matrix material. Wang et al. reported that the repair of articular cartilage defects had been achieved with ASCs and acellular cartilaginous matrix in rabbits [112]. In the tissue-engineered cartilage group (ACM combined with ASCs) after 12 weeks, articular cartilage defects were filled with chondrocyte-like tissue with a smooth surface and were rich in glucan and type II collagen, similar to normal articular cartilage. Although the development of cartilage tissue engineering is still in its infancy, the acellular cartilaginous matrix obtained in this study offers tremendous potential in cartilage regeneration medicine. Recently, a decellularized liver 3D matrix scaffold has been proved to be able to facilitate the activity and function of the hepatic cells and stem cells [113–115]. Zhang and Dong [113] compared the hepatogenic differentiation-inducing effect of decellularized liver 3D matrix scaffold and several extracellular matrices, including collagen, fibronectin, and Matrigel in combination with mouse adipose-derived mesenchymal stem cells* in vitro*. The results clearly demonstrated that decellularized liver ECM gel, either on its own or in the presence of grow factors, could significantly enhance hepatic differentiation from ASCs compared with other matrix scaffolds; this demonstrates the feasibility of liver DCM as a bioscaffold for liver regenerative medicine and tissue engineering. Paper, which is produced from natural sources, can be supplied in large quantities with fair properties of biocompatibility and cost-effectiveness [116]. Hence, paper may have the potential in establishing tissue engineering scaffolds for therapeutic application of stem cells. Park et al. reported the feasibility of a paper-based bioactive scaffold for hASCs application to repair bone tissue defects for the first time [117]. In this study, paper scaffolds were prepared from three types of commercial paper materials: weighing paper (WP), chromatography paper (CP), and wiping tissue (WT), after which a polymer-coating method called initiated chemical vapor deposition (iCVD) was employed to coat the paper scaffold to achieve favorable biochemical surface properties (e.g., adhesiveness and water resistance), without damaging the scaffolds. The results showed that osteogenic differentiation of hASCs was induced on the paper scaffolds under osteogenesis-inducing conditions* in vivo*, indicating that paper material possesses great potential as a bioactive, functional, and cost-effective natural scaffold for adipose stem cell-mediated bone tissue engineering. Insoluble (derivatized or crosslinked) forms of HA have been extensively investigated for tissue engineering purposes due to HA's role in the extracellular matrix as well as its biocompatibility, nonimmunogenicity, high hygroscopicity, and capacity to degrade into safe products [118–121]. Desiderio et al. evaluated the differentiation potential of constructs made from a new crosslinked HA (XHA) scaffold on which NG2+ ASCs were loaded. Thirty days after engraftment in mice, NG2+ ASCs underwent a complete myogenic differentiation and fabricated human skeletal muscle tissue, indicating a significant step in muscle regeneration without the need for a prior* in vitro* muscle differentiation step [121].

In summary, the application of natural materials in the field of regeneration medicine is currently progressing. The advantages of natural materials are biocompatibility and mechanical and biological properties consistent with* in vivo* features, making them perfect candidates for tissue engineering field. Apart from the neoteric materials mentioned above, other commonly used natural materials in tissue engineering include collagen [122–125], hyaluronan [126], Matrigel [127, 128], and chitosan [129].

## 5. Clinical Applications of Different Carrier Scaffolds in Combination with ASCs in Tissue Engineering Strategies

The major role of regenerative medicine in this century is based on cell therapy, in which ASCs hold a key position [130–135]. Recently, a number of* in vitro* and a few* in vivo* studies using ASCs in combination with carrier scaffolds can be found through searches and on clinical trial websites. However, the use of cultured stem cells in clinical settings is strictly controlled by governmental regulations around the world, which largely restrict the application of ASCs in regenerative medicine. Meanwhile, plastic surgeons in Korea and Japan have played a leading role in pioneering the use of ASCs in tissue engineering more than any other Western nations due to less stringent government regulations.

It is well known that diabetic ulcers and chronic radiation ulcers are notorious for their recurrence, or failure to heal due to patient debilitation or poor local blood supply conditions. Several conventional reconstructive surgeries have been introduced for patients with chronic nonhealing cutaneous lesions [136, 137]. Presently, Kim and Jeong reported a less invasive method using adipose stem cell-based therapy and a collagen sponge scaffold, which was covered with an artificial dermis (Terudermis®) to deal with a chronic diabetic ulcer on the knee area. Two weeks after application, vascular tissue ingrowth was seen in the lesion area and thus a skin graft could be placed on the newly engineered vascular bed [5]. Complex fistulas are difficult to manage. Currently limited surgical procedures often result in high recurrence rates, whereas extensive surgical procedures may cause fecal incontinence. One recent improvement in treating complex fistulas may be the use of ASCs in combination with a fibrin glue scaffold described by Garcia-Olmo et al. [138]. The fibrin glue used in this study contained human fibrinogen, bovine aprotinin, and human thrombin, and the ASCs were isolated from lipoaspirated fat tissue. Eight weeks after the final treatment, fistula healing was observed in 17 (71 percent) of 24 patients who received fibrin glue plus ASCs, in comparison to 4 (16 percent) of 25 patients who received fibrin glue alone. The proportion of patients with healing strongly indicates that the combination of the fibrin glue scaffold and ASCs is an effective and safe treatment for complex perianal fistulas. Autogenous bone graft has been considered to be the gold standard for reconstructive bone surgery. However, harvesting bone for grafting is associated with significant donor site morbidity that requires additional operative and anesthetic time [139]. An alternative approach is bone tissue engineering, through which in situ bone formation by using combinations of biomaterials, bioactive molecules, and stem cells can be achieved [140, 141]. Sandor et al. reported a case that used an ASC tissue-engineered construct to treat a large anterior mandibular defect. In this report, expanded ASCs were seeded on *β*-tricalcium phosphate (*β*-TCP) granular scaffolds consisting of recombinant human bone morphogenetic protein-2; the constructs were implanted into a U-shaped titanium mesh that spanned the parasymphyseal defect. Ten months after reconstruction, dental implants were integrated into the grafted site successfully with a dental implant-supported overdenture. The patient has been followed for three years since the tissue-engineered constructs were placed; he has been pleased with the aesthetic outcome of the procedure and continues to be satisfied with the function of his dental implants.

Although the applications of scaffolding materials together with ASCs technologies are a rapidly developing field of regeneration medicine, they are highly experimental so far. Thus, there still remains a significant need to develop efficient carrier materials that may bridge the gap and lead towards clinical applications in tissue engineering.

## 6. Conclusions and Future Perspectives

In the past several years, evidence has demonstrated that the ECM not only offers structural support for cells but also profoundly influences the major cellular programs of growth, differentiation, and apoptosis [142, 143]. An ideal scaffold structure must accomplish the roles of the extracellular matrix for the seeded cells, which will be used to form a tissue-engineered construct and to promote the repair/regeneration of damaged tissue [144, 145]. Hence, the design of carrier materials that can regulate cell behaviors such as proliferation and differentiation is the main purpose for the fabrication of tissue engineering scaffolds. Moreover, we should begin to understand that biomaterials and scaffolds used in tissue engineering strategies are dynamic, mobile, and multifunctional regulators of cellular behavior, and not just mere carriers for stem cells or storehouses for cytokines. Indeed, as the field of tissue engineering is still in its infancy, possible biomechanics and biomechanical effects created by different types of scaffolds on seeded ASCs should be further elucidated so that carrier scaffolds with special properties can be created. The creation of such scaffolds would help us to optimize cellular activities including changes in morphology, proliferation, and differentiation.

In general, carrier materials for the successful generation and maintenance of engineered tissue constructs should have several necessary properties including biocompatibility, degradability, low toxicity, and immunogenicity. However, most carrier scaffolds possess only some of these desirable properties. If a scaffold could embody all of these properties successfully, it would provide an ideal platform for tissue regeneration.

In conclusion, ASCs have a prominent and strong role in tissue engineering and regenerative medicine due to their high cell yield in adipose tissue, their ability to differentiate into multiple lineages and secrete various cytokines, and their immunomodulatory effects. The field of ASCs-based tissue engineering therapy is still young. Ongoing and future development of carrier scaffolds together with reasonable promotion of stem cell research and clinical studies will no doubt gradually bring ASC-based tissue engineering technology down from the ivory tower and make it clinically accessible on a larger scale, thereby benefiting more patients.

## Figures and Tables

**Table 1:** Synthesis of synthetic materials for tissue engineering applications using ASCs.

Materials	Properties	Principal uses	References
TiO_2_ nanofiber	High degree of crystallinity and surface wettability	Bone tissue engineering	[85]

Poly(3-hydroxybutyrate-co-hydroxyvalerate) (PHBV) scaffold	Good integration with the surrounding tissue, stiff character, and degradability	Skin tissue engineering	[10]

Copolymer PEGylated fibrin (P-fibrin) gels	Stable urethane (carbamate) linkage, degradability	Cardiovascular and skin tissue engineering	[91]

Poly(glycerol sebacate) (PGS)/poly(L-lactic acid) (PLLA) blend scaffolds	Favorable porous microstructures, good hydrophilicity, appropriate mechanical properties for soft tissue applications, and degradability	Adipose tissue engineering	[93]

Poly(lactic-coglycolicacid)/multiwalled carbon nanotubes/silk fibroin (PLGA/MWCNTs/SF) nanofibrous scaffolds	Nonwoven structures and random fiber distribution with smooth and beadless fibers morphology	Nerve tissue engineering	[95]

[Poly-D,L-lactic acid/polyethylene glycol/poly-D,L-lactic acid (PDLLA-PEG)]/hyaluronic acid (HA) matrix	Designed architectures, high mechanical strength and biodegradability, biocompatibility, and water solubility	Cartilage tissue engineering	[99]

**Table 2:** Synthesis of natural materials for tissue engineering applications using ASCs.

Materials	Properties	Principal uses	References
Decellularized human adipose tissue extracellular matrix (hDAM)	Maintains the major adipose tissue ECM components and 3D structure and includes collagen, sulfated glycosaminoglycan, and and vascular endothelial growth factor but lacks major histocompatibility complex antigen I	Adipose tissue engineering	[12]

Acellular cartilage matrices (ACMs)	Ideal 3D structure and physicochemical properties and good biocompatibility	Cartilage tissue engineering	[112]

Liver decellularized extracellular matrix (DCM)	Preserves macroscopic 3D architecture and the native composition, and ultrastructure remains a viscous liquid at low temperatures (at or under room temperature) and becomes gelation at 37°C	Liver tissue engineering	[113]

Paper-based bioactive scaffold	Microfibrous porous 3D architecture and biocompatible, cost-effective, mechanical robustness and water resistance	Bone tissue engineering	[117]

Hyaluronic acid scaffold	Biocompatibility, nonimmunogenicity, high hygroscopicity, and capacity to degrade into safe products	Muscle tissue engineering	[121]

Collagen	Nontoxic, biocompatible, and bioabsorbable, and it is FDA approved for use in humans	Adipose regeneration and adipose tissue engineering	[122, 124, 125]

Matrigel	Natural polymer and biocompatible	Adipose tissue engineering	[128]

Chitosan	Biodegradable, biocompatible, and an excellent hemostatic and analgesic agent with antioxidant properties	Skin reconstruction and skin tissue engineering	[128, 129]
